# Barriers and Facilitators to Psychologists’ Telepsychology Uptake during the Beginning of the COVID-19 Pandemic

**DOI:** 10.3390/ijerph20085467

**Published:** 2023-04-11

**Authors:** Jack D. Watson, Bradford S. Pierce, Carmen M. Tyler, Emily K. Donovan, Kritzia Merced, Margaret Mallon, Aaron Autler, Paul B. Perrin

**Affiliations:** 1 Department of Psychology, Virginia Commonwealth University, Richmond, VA 23284, USA; 2 Central Virginia Veterans Affairs Health Care System, Richmond, VA 23249, USA; 3 School of Data Science and Department of Psychology, University of Virginia, Charlottesville, VA 22903, USA

**Keywords:** telepsychology, telehealth, COVID-19, coronavirus, implementation

## Abstract

The COVID-19 pandemic transformed the delivery of psychological services as many psychologists adopted telepsychology for the first time or dramatically increased their use of it. The current study examined qualitative and quantitative data provided by 2619 practicing psychologists to identify variables facilitating and impeding the adoption of telepsychology in the U.S. at the beginning of the COVID-19 pandemic. The top five reported barriers were: inadequate access to technology, diminished therapeutic alliance, technological issues, diminished quality of delivered care or effectiveness, and privacy concerns. The top five reported facilitators were: increased safety, better access to patient care, patient demand, efficient use of time, and adequate technology for telepsychology use. Psychologists’ demographic and practice characteristics robustly predicted their endorsement of telepsychology barriers and facilitators. These findings provide important context into the implementation of telepsychology at the beginning of the pandemic and may serve future implementation strategies in clinics and healthcare organizations attempting to increase telepsychology utilization.

## 1. Introduction

In mid-March 2020, the World Health Organization (WHO) declared a pandemic caused by a new type of coronavirus (COVID-19; [1]). With details of the transmissibility of the virus unknown at the time, the WHO issued worldwide recommendations for interpersonal distancing to stem the tide of infection [2]. U.S. businesses, government entities, and other institutions responded to the WHO recommendations by making a rapid shift to the utilization of online technologies for conducting their daily business whenever possible [3]. This move to a largely virtual environment included healthcare settings, as the Centers for Disease Control (CDC) and the American College of Surgeons (ACS) refined the WHO’s recommendations further by advising the postponement of elective procedures and routine face-to-face healthcare visits [4,5]). Anticipation of a situation where healthcare systems could become overwhelmed by ever-increasing demands for beds, medical supplies, and care from providers who were themselves at high risk of COVID-19 exposure led large healthcare organizations such as the Veterans Health Administration (VHA) and the Mayo Clinic Health System to swiftly convert to providing the majority of their care with telehealth [6,7,8]. 

Of special concern to healthcare providers is the increase in mental health issues associated with the pandemic. Anxiety, panic disorder, and depression initially rose in response to unknowns about relative risks [9,10], and measures taken to preclude contagion may also have contributed to decreased mental wellbeing [11]. COVID-19-related mental health issues have also been noted in special populations, such as older adults [12] and those who were already receiving mental health services, even to the point of overshadowing original mental health concerns [13]. Additionally, over one-third of the U.S. population lives in areas that are underserved by mental health care services and providers [14]. This escalated need for psychological services in the pandemic milieu has promoted the adoption of telepsychology, defined as the use of any of a variety of telecommunication technologies to provide mental health interventions [15], at a rate much faster than in the prior non-pandemic setting [16]). Telepsychology and in-person sessions are equally effective [17]) and efficient [18,19,20] for treatment of most psychological issues, but prior to the pandemic, telepsychology accounted for only 7% of psychologists’ practice [16].

Reasons for such a small portion of psychological treatment being provided with telepsychology pre-COVID-19 from the psychologists’ perspective include intrapsychic, practice preference, and environmental concerns. Many psychologists cited perceived difficulties in using telepsychology and the lack of training leading to their low self-efficacy regarding the personal use of telepsychology in their practice [16,21,22]. Psychologists may have feared that important data pertaining to client appearance and behavior, such as subtle changes in eye contact and speech modulation, would be lost when not meeting face-to-face with clients [23,24]. Environmental barriers included privacy laws [25], Medicare regulations [26] (O’Reilly, 2019), state residency/license requirements, and practice setting restrictions such as appropriate space, adequate equipment, and the existence of telepsychology policies and procedures [16,22].

Although social distancing requirements brought about by COVID-19 may have seemed onerous when first implemented, the subsequent expansion of telepsychology may have facilitated access to psychological treatment for people who had hitherto been constrained from engagement in face-to-face sessions by transportation/distance, time, disability, caregiving issues [27], or the need to be seen by practitioners with expertise in specific domains who were not previously geographically accessible when in-person meetings were the norm [28,29]. Likewise, telepsychology use has increased patient safety by reducing exposure risks for vulnerable populations which rely on public transportation for getting to and from mental health appointments [13].

Responding to the need for in-person visits to be minimized while maintaining continuity of care during the pandemic, Medicare and Medicaid eased restrictions by reimbursing telepsychology visits at the same rate as in-person visits [30]. Not only has this increased accessibility for those who have Medicare and Medicaid as their insurance plans, it also provides an example for private insurers who look to Medicare to lead the way when changes are in order [31]. Ensuring comparable reimbursement of telepsychology visits has enabled consumers to seek or continue their psychological care without the added burden of worrying about affordability. Another factor vital to enabling psychologists to quickly meet patient mental health needs with telepsychology was the Department of Health and Human Services’ (HHS) Office for Civil Rights’ (OCR) decision to allow commonly used apps such as FaceTime, Zoom, and Skype to be used for telehealth, even though they do not meet Health Insurance Portability and Accountability Act (HIPAA) standards [32,33]. Following the lead of federal entities in easing telehealth rules, many states responded to the public health emergency by lifting restrictions requiring psychologists to be licensed in the state where their patient is located at the time of the appointment and regulations regarding the preexistence of provider–client relationships, telehealth modalities, etc. [34,35,36,37]. 

Government entities have not been the only groups to step up to the challenge of providing means for smoothing an expeditious transition to telehealth. Realizing the immediate need for telepsychology practice guidelines and training, psychology organizations such as the American Psychological Association (APA), the European Federation of Psychologists’ Associations (EFPA), and the Australian Psychological Society (APS) have published resources for using telepsychology for their members and the public [38,39,40].

Although some have pointed out that the use of telepsychology represents the next step in the integration of technological tools already in common use (e.g., intranet, electronic medical records, and videoconferencing) [41], numerous barriers still exist. For example, technological issues such as problems with bandwidth and network latency can interfere with good two-way communication [24]. The adoption of telepsychology as the chief mode of practice with clients throughout the COVID-19 pandemic has compelled psychologists to acquire or improve competency with the technological components necessary for conducting telepsychology. Psychologists must be adept enough with the use of computers or other Internet-capable devices, cameras, microphones, and telecommunications software and applications such that they are able to troubleshoot problems and guide their clients in using the technologies as well [15,16].

Access to telepsychology services differentially affects patients and patient groups. For example, those with lower socioeconomic status may have difficulty affording Internet-capable devices, adequate data plans, or high-speed Internet connections, effectively precluding them from utilizing telepsychology services. Lack of technological expertise, physiological impairments, or communication difficulties present limitations in the use of telehealth by more than one-third of older adults [42]. COVID-19 has highlighted some of the inequities in healthcare access across the U.S. and globally. Given that certain population groups including racial/ethnic minorities, those with chronic health conditions and disabilities, and older adults are at higher risk of severe complications from COVID-19 [43], impediments to the use of telepsychology could have differential, devastating consequences. As telepsychology rates skyrocketed during the pandemic with initial estimates suggesting that more than 85% of psychological services were provided with telepsychology [44], it is important to understand what variables during the pandemic have facilitated or impeded its use, as well as potential clinical implications. Therefore, the current study examined qualitative and quantitative data provided by 2619 practicing psychologists to identify variables facilitating and impeding the adoption of telepsychology in the U.S. at the beginning of the COVID-19 pandemic. Further, psychologists’ personal and practice demographics were collected to help identify which characteristics were associated with the likelihood to endorse a particular barrier or facilitator to telepsychology uptake.

## 2. Method

### 2.1. Participants

Psychologists were invited by email to participate in the current study using a list of email addresses gathered from publicly available information on websites for mental health services, psychology newsgroups, and membership rosters within professional organizations. Participants were sent an initial email invitation with a reminder seven days later. Responses were collected over two weeks from 11 May 2020 to 25 May 2020. Eligibility criteria required that participants were (a) licensed to practice as a psychologist in the U.S., (b) aged 18 or older, and (c) currently practicing (seeing patients) as a psychologist in the U.S. A total of 27,324 invitations were sent with 3897 (14.3%) returning as undeliverable. Of those remaining, 3694 (15.8%) people opted to review the information; 184 of those left after viewing the information or did not provide informed consent; 541 left the survey after providing consent; 198 were not eligible because they did not treat patients; 127 did not answer demographic questions; and 25 responded illogically to the question, “In one sentence or less, what do you think the purpose of this study was?” This process left a final sample of 2619 participants (Table 1) completing the survey, resulting in an 11.2% response rate. This response rate is typical for studies using similar recruitment techniques [44,45]. Data for the current study were collected as part of a larger study analyzing environmental and demographic predictors of psychologists’ use of telepsychology during the COVID-19 pandemic [46]. This study was approved by the Virginia Commonwealth University Institutional Review Board (IRB) to ensure it was conducted ethically and in compliance with all federal, state, and local regulations concerning research involving human participants.

### 2.2. Procedure

This study is part of a secondary data analysis of a larger study on telepsychology use during the COVID-19 pandemic [46]. Emailed invitations included a brief description of this study and an HTML link to the informed consent page. The online consent page provided more details about this study, IRB information, and methods for contacting the authors. The invitation and consent page were crafted to provide sufficient information about this study without specifically mentioning telehealth or telepsychology in order to reduce the possibility of bias toward telepsychology influencing those who chose to participate. Those who consented were then redirected to the survey. The first three items asked if participants were currently licensed to practice as a psychologist within the U.S., if they were currently practicing (seeing clients/patients), and their current age. Those who met the eligibility criteria were then asked a set of demographic questions about themselves and their primary place of practice. They were also provided a definition stating, “for the purpose of this survey, ‘telepsychology’ refers to the use of real-time audio (e.g., telephone) and/or video conferencing technology to provide psychological services”.

### 2.3. Measures

Personal and Practice Demographics. Participants were asked to provide their age, years in practice, gender, race/ethnicity, the population density of their practice location (e.g., urban, suburban, or rural), primary treatment setting, the number of psychologists practicing within their primary treatment setting, their theoretical therapeutic approach(s) to practice, the treatment focus of their setting, if they had ever used telepsychology, and the percentage of their patient treatment that they currently provided using telepsychology.

Advantages and Incentives. Participants were asked to select any “advantages or incentives for using telepsychology in your practice to treat patients”. Choices included “better patient access to care”, “lower costs for patients”, “lower costs for my practice”, “telepsychology training I have received”, “better therapeutic alliance”, “efficient use of time”, “effectiveness”, “leadership support”, “higher quality of delivered care”, “ethics”, “supportive HIPAA regulations”, supportive laws”, “patient demand or preference for telepsychology”, “increased safety (e.g., limiting contagion)”, and “adequate video/audio quality of telepsychology technology”. Participants were allowed to select multiple items and were also provided a free response option labeled “Other (Please specify)” to indicate any additional advantages and incentives not listed.

Barriers and Deterrents. Participants were asked to select any “barriers to or deterrents against using telepsychology in your practice to treat patients”. Choices included “lack of patient access to technology”, “insufficient insurance reimbursement”, “higher costs for patients”, “higher costs for my practice”, “insufficient telepsychology training, “diminished therapeutic alliance”, “inefficient use of time”, “lack of leadership support”, “diminished quality of delivered care”, “lack of patient demand for telepsychology”, “ethical issues”, “HIPAA requirements”, “legal issues”, “unsupportive prescription regulations”, “higher risk to patient safety”, “higher risk to patient privacy”, and “inadequate video/audio quality of telemedicine technology”. Participants were allowed to select multiple items and were also provided a free response option labeled “Other (Please specify)” to indicate any additional barriers and deterrents not listed.

The selectable options for Advantages/Barriers were researcher generated from the previous literature on telepsychology barriers, facilitators, and the likelihood of use [16,46,47]. Further, the collected demographic and practice data were selected from previous research that found those specific characteristics to be potentially important in predicting telepsychology uptake [47].

## 3. Results

### 3.1. Descriptive Analyses

We utilized a qualitative analysis process described by Zhang and Wildemuth [48] to code the “Other [Please specify]” options for both advantages/incentives and barriers/deterrents. Using the initial set of codes and descriptors provided in the survey, two raters familiar with the telepsychology literature reviewed the initial 669 “Other [Please specify]” responses (249 advantages/incentives and 420 barriers/deterrents). The two raters independently sample-coded a random selection of items, resulting in an interrater reliability of less than 50%. The descriptors for the thematic categories were modified for greater clarity by the two raters. Additionally, for “Other [Please specify]” responses that had two distinct themes, a third researcher separated these into individual responses, generating 686 separate thematic responses. As an example, if a participant checked “Other [Please specify]” and wrote two distinct responses such as (a) “Telepsychology is cheaper for me” and (b) “It’s safer for health compromised patients”, these were separated into two distinct answers to fit separate thematic categories. The first two raters then independently sample-coded an additional selection of items, resulting in an interrater reliability of 67%.

For barriers to telepsychology use, the first two raters then combined two pairs of categories: (a) higher patient cost and higher provider cost were merged into one category called “higher cost for patient or provider” and (b) legal issues and HIPAA requirements were merged into “legal and HIPAA concerns”. Additionally, we created three categories: “telepsychology is incompatible with certain populations”, “telepsychology is incompatible with certain services”, and “miscellaneous”. These categories reflected thematic responses to the “Other [Please specify]” category that were not represented in the researcher-generated list of categories.

For facilitators to telepsychology use, we combined three pairs of categories: (a) lower cost to patient and lower cost to provider were combined into one category called “lower cost for patient or provider”, (b) supportive laws and supportive HIPAA regulations were merged into “supportive laws and HIPAA regulations”, and (c) effectiveness and higher quality of delivered care were combined into “Improved quality of delivered care or effectiveness”. Additionally, we added two categories: “provider preference for telepsychology” and “miscellaneous”. Again, these categories reflected thematic responses to the “Other [Please specify]” category that were not represented in the researcher-generated list of categories.

This resulted in a final list of 19 barriers to telepsychology use and 15 facilitators (Table 2). The two raters coded a new set of data, achieving an interrater reliability of greater than 75%, and then coded the remaining items. A total of 649 “Other [Please specify]” responses were recoded into the 24 categories, as 37 responses were removed from the dataset due to nonsensical, irrelevant, or uninterpretable content. Example responses that were removed included: “None”, “Unclear at this time”, “None of the above”, “Usually a barrier but sometimes not”, “No real barriers”, and “COVID only”. A fourth researcher, also familiar with the telepsychology literature, served as a tiebreaker for any items on which the first two coders disagreed, choosing only one category from those endorsed by the original two raters. The final, tie-broken list was then recombined with the list of responses obtained from the provided options and analyzed. We summarized the barriers and facilitators to telepsychology use by calculating the percentage of psychologists who endorsed each option (Table 3).

### 3.2. Logistic Regression

We ran a series of logistic regressions to predict, using participant demographics and practice characteristics, the most frequently endorsed (20% or more) barriers (Table 4) and facilitators (Table 5) with not endorsing the barrier or facilitator as the reference category. Predictors included the continuous variable age and binary variables woman gender (vs. men and others), White/European American race/ethnicity vs. racial/ethnic minorities, practicing within a rural community vs. urban or suburban, practicing within a medical center vs. other settings, and using cognitive behavioral therapy (CBT) as a treatment approach to therapy vs. other approaches. All models except two (the facilitators Adequate Technology for Telepsychology Use and Improved Quality of Delivered Care or Effectiveness) were statistically significant, χ^2^(6) > 12.91, *p*s < 0.05, and those that were had a Nagelkerke pseudo-*R*^2^ range from 0.1 to 3.6%.

### 3.3. Barriers

Older psychologists were less likely to report barriers to telepsychology use such as technological issues, diminished quality of delivered care or effectiveness, privacy concerns, inadequate reimbursement, insufficient telepsychology training, ethical concerns, and safety concerns. Cisgender women were less likely to report barriers such as diminished therapeutic alliance, technological issues, diminished quality of delivered care or effectiveness, and legal and HIPAA concerns, but they were more likely to report privacy concerns. White psychologists reported all barriers at the same rate as those from racial/ethnic minority backgrounds. Rural psychologists were more likely to report barriers such as inadequate access to technology and ethical concerns. Psychologists working in medical centers were more likely to report barriers such as inadequate access to technology, but they were less likely to report diminished therapeutic alliance and privacy concerns. Psychologists who practiced with CBT were more likely to report barriers such as inadequate access to technology and inadequate reimbursement, but they were less likely to report diminished quality of delivered care or effectiveness.

### 3.4. Facilitators

Older psychologists were less likely to report facilitators to telepsychology use such as better access to patient care and patient demand. Cisgender women were more likely to report facilitators such as increased safety, supportive laws and HIPAA regulations, and improved quality of delivered care or effectiveness. White psychologists were more likely to report the facilitator as patient demand, but they were less likely to report efficient use of time and lower cost for patient or provider. Rural psychologists were less likely to report the facilitator as patient demand. Psychologists working in medical centers were more likely to report facilitators such as better access to patient care and lower cost for the patient or provider, but they were less likely to report supportive laws and HIPAA regulations. Psychologists who practiced with CBT were more likely to report facilitators such as better access to patient care, patient demand, efficient use of time, and lower cost for the patient or provider.

## 4. Discussion

This study examined qualitative and quantitative data provided by 2619 practicing psychologists to identify variables facilitating and impeding the adoption of telepsychology in the U.S. at the beginning of the COVID-19 pandemic. The top five reported barriers were inadequate access to technology, diminished therapeutic alliance, technological issues, diminished quality of delivered care or effectiveness, and privacy concerns. The top barriers to telepsychology use at the beginning of the COVID-19 pandemic found in this study are broadly consistent with findings in other studies during this time with other healthcare providers [49,50,51]. For instance, a qualitative study exploring the transition of 20 psychiatrists to telemedicine at the beginning of the pandemic found similar concerns related to patient privacy, disparities in access to reliable technology for visits, and concerns about the quality of delivered care [49]. A larger qualitative study examining 934 mental health professionals’ views at the beginning of the pandemic found similar barriers including concerns about its effectiveness, problems with technology use (i.e., poor connectivity and video quality), and worries about replicating the therapeutic environment [52]. The current findings provide additional context to these previous studies by exploring demographic and professional characteristic predictors for the endorsement of these barriers and facilitators. This is particularly important as it may highlight potential explanations for patterns in engagement with telepsychology across groups and settings.

In the current study, older psychologists reported fewer barriers than younger psychologists in terms of technological issues, diminished quality of delivered care, privacy concerns, inadequate reimbursement, insufficient training, and ethical and safety concerns, potentially pointing to alternative reasons for their engagement patterns. This finding is unlikely to be due to age differences in telepsychology use at the beginning of the pandemic [46]. Instead, a possible interpretation is that older, and therefore more seasoned psychologists, may have more experience or a more established caseload than younger psychologists and, therefore, may have had training that served to navigate some of these barriers.

Women psychologists were more likely to report barriers related to technology and privacy concerns, but they were less likely to report barriers related to the therapeutic alliance, legal/HIPAA concerns, or quality and effectiveness of delivered care. These findings are generally in line with socialized gender norms, such that women may perceive stronger interpersonal connections to others yet can also be acutely aware of technology barriers and safety-related concerns [53]. Thus, it is possible that internalized beliefs about women and lower technological competence (or conversely, men believing they are more technologically competent) may influence their own views of telepsychology barriers. Additionally, women may have had additional caregiving responsibilities at home, highlighting privacy concerns (e.g., caretaking for a child interfering with work privacy).

Providers who practiced or served in rural areas were more likely to report barriers related to inadequate access to technology and ethical concerns, which is consistent with the reported challenges in bringing adequate internet and access to many rural communities in the U.S. [54]. Additionally, psychologists serving a rural client population using telepsychology may have had ethical concerns about the ability to marshal emergency services or follow-up services if needed, given other limitations in access to care in rural regions.

Psychologists practicing CBT were more likely to report barriers related to inadequate access to technology and inadequate reimbursement; these providers were also less likely to report barriers related to diminished quality of delivered care or effectiveness. It is possible that CBT-using psychologists had been accustomed to sharing worksheets and assigning and explaining structured homework assignments, so inadequate access to technology (e.g., computers, smartphones) could prevent full adherence to many evidence-based CBT treatments. Psychologists using CBT may also have been accustomed to billing insurance companies for very specific evidence-based approaches that may not be as readily usable with telepsychology as unstructured therapy or other therapeutic approaches. At the same time, psychologists practicing CBT may have felt more confident in the effectiveness of their interventions given the evidence base of CBT [55].

The top five reported facilitators were increased safety, better access to patient care, patient demand, efficient use of time, and adequate technology for telepsychology use. Overall, the top facilitators in this study align with other studies [52] and highlight how these facilitators may be used to encourage further utilization in the modality, expansion in services, and policy changes [50] beyond the pandemic and more as a permanent tool for mental health services. Given the COVID-19 pandemic, it is somewhat unsurprising that the top facilitators of telepsychology use were increased safety to prevent the spread of COVID-19 and better access to patient care during the initial phase of the lockdown period; it is also not surprising that there would be high patient demand for telepsychology, considering the improved safety and access. Older psychologists were less likely to report better access to patient care and patient demand as facilitators; however, these effects were extremely small and were likely statistically significant due to this study’s large sample size. Women psychologists were more likely to report facilitators related to increased safety, supporting laws and HIPAA regulations, and improved quality of delivered care or effectiveness when compared to men and transgender/non-binary psychologists. The pattern of women being more likely to endorse these three facilitators might be reflective more holistically of women’s openness to using telepsychology during the beginning of the COVID-19 pandemic [46] given its practical benefits when juggling a greater number of household and childcare responsibilities than men [56].

White psychologists were more likely to report facilitators related to patient demand and less likely to report facilitators related to efficient use of time and lower cost for the patient or provider. This finding contrasts with the lack of an effect of psychologist race on any reported barrier to using telepsychology. Although this interpretation is speculative, White psychologists might tend to serve a higher-income clientele with greater fluency in telehealth approaches, therefore generating more perceived patient demand for telepsychology services in their caseload and particularly in patients who might be able to pay out of pocket (therefore, not seeing lower cost as a facilitator). The reason for the effect of the psychologist’s White race on being less likely to endorse efficient use of time as a facilitator is unknown and a direction for future research; however, it is possible that White psychologists may not have perceived a significant difference in the use of time between in-person and telepsychology modalities.

Rural-practicing psychologists were less likely to report facilitators related to patient demand. This finding was just statistically significant (*p* = 0.048) but nonetheless in line with the findings noted above that rural psychologists were more likely to report barriers related to inadequate access to technology and ethical concerns. As a result, patients served by rural psychologists may not have had the technological access to create larger telepsychology demand.

Psychologists in medical centers were more likely to report facilitators related to better access to patient care and lower cost for patients and providers, but they were less likely to report facilitators related to supportive laws and HIPAA regulations. Given that many medical centers are in larger cities, medical center psychologists may have been better able to serve patient populations unable to travel to the medical center due to the lockdown in the early part of the COVID-19 pandemic, which may have been stricter in larger cities. Additionally, this may have saved money for medical center patients who might tend to have lower incomes than patients served in private practices. Conversely, medical centers may have been slower to adapt to new laws and HIPAA regulations loosening restrictions on telepsychology practice than less bureaucratic private practices or small practice settings.

Psychologists practicing CBT were more likely to report facilitators related to better access to patient care, patient demand, efficient use of time, and lower cost for patient or provider. This pattern in the findings has not emerged in the known literature to date and does not have an easily interpretable cause; as a result, future research should explore this pattern in order to shed light on the patterns of telepsychology facilitators in CBT-practicing psychologists. 

### Limitations and Future Directions

Despite this study identifying the barriers and facilitators to the adoption of telepsychology at the beginning of the COVID-19 pandemic, it has some limitations. First, the data were collected at the beginning of the pandemic, and thus, potentially limit consideration of the context and changes influencing psychologists’ telepsychology adoption at later stages of the pandemic and beyond. Since the beginning of the pandemic, there were many barriers and facilitators that likely impacted psychologists’ use of telepsychology, including macro level changes in the state parity laws, social distance mandates, reimbursement, and HIPAA laws. A potential follow-up study could explore current barriers and facilitators to adopting telepsychology to see if there has been a significant change. Further research may seek to develop and test a hybrid in-person/telepsychology practice model, with greater consideration for the current state of COVID-19 social restrictions, vaccination rates, and increase in mental health apps and telepsychology platforms (e.g., Cerebral, Better Help, Doximity, etc.).

A second limitation to this study involves the nature of the qualitative design developed by Zhang and Wildermuth [48], which attempts to preserve the methodological strengths of qualitative coding and quantitative content analysis. However, it is possible that the approach was inherently reductive and may have lost depth in the participants’ responses. Future research may add to the literature by using more phenomenological approaches such as focus groups and in-depth interviews. This could expand the interpretation of the current findings by adding nuanced context to the barriers and facilitators.

Lastly, underrepresentation may have occurred for some facilitators and barriers. There were multiple categories added during the qualitative analyses process based on typed responses to the “other” category. Though the “other” category was endorsed multiple times, and participants included a written response, and it is possible that these categories would have been endorsed more frequently if they had initially been included in the checklist.

## 5. Conclusions

This study examined qualitative and quantitative data provided by 2619 practicing psychologists to identify variables facilitating and impeding the adoption of telepsychology in the U.S. at the beginning of the COVID-19 pandemic. Psychologists endorsed inadequate access to technology as the most frequent barrier to telepsychology and increased safety as the most frequent facilitator. Regressions models identified specific psychologist demographic and practice-related predictors for the endorsement of these barriers and facilitators. The perceived barriers and facilitators to telepsychology use at the beginning of the COVID-19 pandemic found in the current study—and their demographic and practice-related predictors—may help health systems and policy makers understand why telepsychology is not being used where that is the case, or conversely, why it is more likely in certain settings. For example, since inadequate access to technology was the most frequently endorsed barrier to telepsychology use, if health systems or providers can provide patients with appropriate technology—and policies support that provision and its reimbursement—telepsychology use will likely be greatly facilitated. Similarly, since increased safety was the most frequently endorsed facilitator to telepsychology use, in various future phases of the COVID-19 pandemic, increased use of telepsychology will likely continue to facilitate appropriate social isolation of infected individuals. These results also support maintaining policies and regulations supportive of telepsychology use (e.g., PSYPACT, Medicare changes, etc.) to facilitate greater access to patient care, which was the second major facilitator of telepsychology use. Overall, these findings provide important context into the implementation of telepsychology at the beginning of the pandemic and may serve future implementation strategies in clinics and healthcare organizations attempting to increase telepsychology utilization.

## Figures and Tables

**Table 1:** Summary of Participant Characteristics.

Characteristics		
Age, *M*, *SD*	57.29	11.42
Years in Practice, *M*, *SD*	24.22	11.03
Gender, *N*, %		
Woman	1681	64.2
Man	928	35.4
Genderqueer	3	0.1
Gender Non-conforming	3	0.1
Transman	2	1.0
Transwoman	1	0.0
Intersex	1	0.0
Race/Ethnicity, *N*, %		
White/European-American	2385	91.1
Latinx/Hispanic	61	2.3
Asian/Asian-American (NH/NL)	50	1.9
Black/African-American (NH/NL)	50	1.9
Multiracial/Multiethnic	40	1.5
Other	30	1.1
American Indian/Alaska Native/Native American	3	0.1
Primary Practice Setting, *N*, %		
Individual Practice	1424	54.4
Group Practice	397	15.2
Hospital/Medical Center	178	6.8
Academic Medical Center	126	4.8
Other	116	4.4
School/University	113	4.3
Outpatient Treatment Facility	99	3.8
Veterans Affairs Medical Center	80	3.1
Geriatric Facility	23	0.9
Psychiatric Hospital	20	0.8
Correctional Facility	19	0.7
Rehabilitation Center	13	0.5
Residential Treatment Center	11	0.4
Practice Location, *N*, %		
Suburban	1229	46.9
Urban	1150	43.9
Rural	240	9.2
Number of Psychologists in Practice, *N*, %		
1	1177	44.9
2–5	864	33.0
6–10	246	9.4
11–20	182	6.9
21–50	82	3.1
50+	68	2.6

**Table 2:** Final List of Barriers and Facilitators.

Barriers	
Code	Description
Higher Cost for Patient or Provider	Concern for financial costs associated with obtaining, implementing, and maintaining telepsychology equipment
Legal and Health Insurance Portability and Accountability Concerns	Statements regarding the legality of telepsychology or HIPAA regulations governing its use
Inadequate Access to Teletechnology	Concern for inability to access technology necessary for telepsychology (e.g., lack of high-speed Internet in rural locations or inability to purchase a smartphone/laptop)
Inadequate Reimbursement	Concern for inadequate reimbursement for telepsychology services from insurance, Medicare, etc.
Insufficient Telepsychology Training	Statements endorsing a lack of training in telepsychology services or policies and procedures
Diminished Therapeutic Alliance	Concern for how the lack of in-person interaction might affect the therapeutic alliance
Inefficient Use of Time	Statements regarding wasted time (e.g., setting up or troubleshooting telepsych issues)
Lack of Leadership Support for Telepsychology	Statements regarding a general dislike/distrust of telepsychology by one’s employer
Diminished Quality of Delivered Care or Effectiveness	Includes concerns regarding efficacy, effectiveness, and the need for more research on telepsychology
Lack of Patient Demand	Statements regarding patients either disliking telepsychology or preferring to meet in person
Provider Dislike of Telepsychology	Statements indicating a general dislike of telepsychology without giving a specific reason
Ethical Concerns	Statements questioning the ethicality of telepsychology
Safety Concerns	Expressions of worry for remote clients who may be in crisis or at risk of harm to self or others
Privacy Concerns	Expressions of worry for remote clients who may not have a private space to engage in therapy or inability for the provider to verify privacy, and also includes concerns over someone hacking into the session
Technological Issues	Includes statements that the technological requirements for telepsychology are currently inadequate or unreliable (e.g., poor connectivity or quality) as well as statements regarding the need for troubleshooting that do not reference “time”
Incompatible with Certain Services	Statements questioning the efficacy of telepsychology for a specifically named services (e.g., play therapy, neuropsychological testing, learning disability testing)
Incompatible with Certain Populations	Statements questions the efficacy of telepsychology for a specifically named populations (e.g., young children, older adults, inmates)
Unsupportive Prescription Regulations	Statements endorsing difficulty navigating prescriptive regulations or stating the prescription regulations are too burdensome
Miscellaneous	Any statements that did not fit in a previously listed category
Facilitators	
Better Patient Access to Care	Statements regarding how telepsychology allows providers to assist patients who would otherwise not be able to be seen
Lower Cost for Patient or Provider	Statements regarding reduced cost for the patient or provider or increased revenue for the provider
Telepsychology Training I Have Received	Statements in which the provider endorsed comfortability with telepsychology as a specific result of having been trained in telepsychology services
Better Therapeutic Alliance	Statements regarding how telepsychology positively effects the therapeutic alliance
Efficient Use of Time	Statements regarding how telepsychology saves either the patient or provider time
Improved Quality of Delivered Care or Effectiveness	Statements indicating better quality or more effective care using telepsychology and statements regarding the growth of research on the efficacy of telepsychology
Leadership Support for Telepsychology	Statements indicating a provider’s employer supports the use of telepsychology
Ethics	Statements indicating that telepsychology is ethically appropriate or imperative
Supportive Laws or Health Insurance Portability and Accountability Regulations	Statements indicating generally positive or supportive laws and HIPAA regulations for telepsychology use
Patient Demand for Telepsychology	Statements indicating a patient’s preference for telepsychology
Provider Preference for Telepsychology	Statements indicating a provider’s preference for telepsychology
Increased Safety for Patient or Provider	Statements indicating an increase in patient or provider safety as a direct result of telepsychology
Adequate Technology for Telepsychology Use	Statements regarding that the technological requirements for telepsychology are currently adequate
Supportive Prescription Regulations	Statements indicating the supportive nature of prescription regulations
Miscellaneous	Any statements that did not fit in a previously listed category

Note. The left-hand column denotes the possible categories into which the “Other” responses were coded. The right-hand column is the operational descriptor used by coders to help decide how best to code an “Other” response.

**Table 3:** Percentage Endorsed for Each Telepsychology Barrier and Facilitator.

Barrier	Percentage
Frequently Endorsed (above 20%)	
Inadequate Access to Technology	62.5
Diminished Therapeutic Alliance	53.3
Technological Issues	49.2
Diminished Quality of Delivered Care or Effectiveness	43.8
Privacy Concerns	42.5
Inadequate Reimbursement	37.5
Legal and Health Insurance Portability and Accountability Concerns	30.2
Insufficient Telepsychology Training	23.9
Ethical Concerns	20.4
Safety Concerns	20.2
Moderately Endorsed (10–20%)	
Lack of Patient Demand	18.6
Higher Cost for Patient or Provider	10.3
Infrequently Endorsed (below 10%)	
Inefficient Use of Time	6.3
Lack of Leadership Support for Telepsychology	4.6
Incompatible with Certain Services	4.0
Unsupportive Prescription Regulations	2.0
Incompatible with Certain Populations	1.9
Miscellaneous	0.5
Provider Dislike of Telepsychology	0.2
Facilitator	Percentage
Frequently Endorsed (above 20%)	
Increased Safety	84.5
Better Access to Patient Care	79.3
Patient Demand	64.5
Efficient Use of Time	56.3
Adequate Technology for Telepsychology Use	42.0
Supportive Laws and Health Insurance Portability and Accountability Regulations	32.8
Lower Cost for Patient or Provider	32.0
Improved Quality of Delivered Care or Effectiveness	24.6
Moderately Endorsed (10–20%)	
Telepsychology Training I Have Received	18.7
Ethics	13.7
Leadership Support for Telepsychology	10.1
Infrequently Endorsed (below 10%)	
Better Therapeutic Alliance	4.0
Miscellaneous	0.7
Provider Preference for Telepsychology	0.0
Supportive Prescription Regulations	0.0

**Table 4:** Variables in the Logistic Regressions for Barriers.

						95% CI for Exp(B)	
Predictor	β	SE	Wald	*p*	Exp(B)	Lower	Upper
Inadequate Access to Technology							
Age	0.002	0.004	0.340	0.560	1.002	0.995	1.010
Cisgender Woman	0.029	0.088	0.111	0.739	1.030	0.866	1.225
White	−0.121	0.146	0.681	0.409	0.886	0.666	1.180
Rural	0.660	0.157	17.686	0.000	1.935	1.423	2.632
Medical Center	0.959	0.183	27.463	0.000	2.610	1.823	3.737
CBT	0.335	0.089	14.058	0.000	1.398	1.173	1.666
Diminished Therapeutic Alliance							
Age	−0.005	0.004	2.184	0.139	0.995	0.987	1.002
Cisgender Woman	−0.228	0.085	7.115	0.008	0.796	0.674	0.941
White	0.121	0.139	0.766	0.381	1.129	0.860	1.481
Rural	0.155	0.138	1.261	0.261	1.167	0.891	1.529
Medical Center	−0.515	0.149	11.929	0.001	0.598	0.446	0.800
CBT	−0.126	0.088	2.049	0.152	0.882	0.743	1.047
Technological Issues							
Age	−0.014	0.004	15.263	0.000	0.986	0.979	0.993
Cisgender Woman	0.244	0.085	8.212	0.004	1.276	1.080	1.507
White	0.157	0.139	1.284	0.257	1.171	0.891	1.537
Rural	0.085	0.137	0.385	0.535	1.089	0.833	1.423
Medical Center	0.113	0.148	0.578	0.447	1.119	0.837	1.496
CBT	0.014	0.088	0.026	0.872	1.014	0.854	1.204
Diminished Quality of Delivered Care or Effectiveness							
Age	−0.008	0.004	5.043	0.025	0.992	0.985	0.999
Cisgender Woman	−0.294	0.086	11.847	0.001	0.745	0.630	0.881
White	0.273	0.143	3.677	0.055	1.314	0.994	1.738
Rural	0.032	0.138	0.055	0.815	1.033	0.789	1.352
Medical Center	−0.153	0.150	1.036	0.309	0.858	0.639	1.152
CBT	−0.360	0.088	16.794	0.000	0.698	0.588	0.829
Privacy Concerns							
Age	−0.012	0.004	10.937	0.001	0.988	0.981	0.995
Cisgender Woman	0.252	0.087	8.486	0.004	1.287	1.086	1.525
White	−0.027	0.140	0.037	0.847	0.973	0.740	1.280
Rural	0.134	0.138	0.944	0.331	1.143	0.873	1.497
Medical Center	−0.601	0.158	14.465	0.000	0.548	0.402	0.747
CBT	0.061	0.089	0.465	0.495	1.062	0.893	1.264
Inadequate Reimbursement							
Age	−0.007	0.004	3.899	0.048	0.993	0.985	1.000
Cisgender Woman	0.168	0.088	3.599	0.058	1.183	0.994	1.406
White	0.091	0.144	0.398	0.528	1.095	0.826	1.451
Rural	0.118	0.139	0.716	0.397	1.125	0.856	1.479
Medical Center	0.148	0.149	0.979	0.322	1.159	0.865	1.553
CBT	0.336	0.092	13.236	0.000	1.399	1.168	1.676
Legal and HIPAA Concerns							
Age	−0.006	0.004	2.514	0.113	0.994	0.986	1.001
Cisgender Woman	−0.189	0.092	4.247	0.039	0.828	0.691	0.991
White	−0.264	0.145	3.318	0.069	0.768	0.578	1.020
Rural	−0.011	0.148	0.006	0.939	0.989	0.740	1.321
Medical Center	0.050	0.158	0.100	0.752	1.051	0.771	1.432
CBT	0.149	0.096	2.389	0.122	1.161	0.961	1.402
Insufficient Telepsychology Training							
Age	−0.011	0.004	7.018	0.008	0.989	0.981	0.997
Cisgender Woman	−0.190	0.099	3.687	0.055	0.827	0.682	1.004
White	−0.206	0.155	1.775	0.183	0.814	0.601	1.102
Rural	0.264	0.151	3.050	0.081	1.303	0.968	1.753
Medical Center	−0.004	0.171	0.001	0.980	0.996	0.713	1.391
CBT	0.024	0.103	0.053	0.819	1.024	0.837	1.253
Ethical Concerns							
Age	−0.019	0.005	17.830	0.000	0.981	0.972	0.990
Cisgender Woman	−0.084	0.106	0.623	0.430	0.920	0.748	1.132
White	−0.202	0.162	1.552	0.213	0.817	0.595	1.123
Rural	0.423	0.155	7.415	0.006	1.527	1.126	2.070
Medical Center	−0.124	0.184	0.452	0.501	0.883	0.616	1.268
CBT	−0.017	0.109	0.025	0.875	0.983	0.793	1.218
Safety Concerns							
Age	−0.014	0.005	9.765	0.002	0.986	0.977	0.995
Cisgender Woman	0.086	0.107	0.640	0.424	1.089	0.883	1.344
White	0.103	0.174	0.349	0.555	1.108	0.788	1.558
Rural	0.225	0.162	1.938	0.164	1.253	0.912	1.721
Medical Center	0.094	0.176	0.284	0.594	1.098	0.778	1.551
CBT	−0.037	0.109	0.113	0.736	0.964	0.778	1.194

**Table 5:** Variables in the Logistic Regressions for Facilitators.

						95% CI for Exp(B)	
Predictor	β	SE	Wald	*p*	Exp(B)	Lower	Upper
Increased Safety							
Age	−0.004	0.005	0.692	0.405	0.996	0.986	1.006
Cisgender Woman	0.595	0.113	27.570	0.000	1.813	1.452	2.265
White	0.315	0.180	3.067	0.080	1.370	0.963	1.948
Rural	−0.033	0.184	0.032	0.858	0.968	0.675	1.388
Medical Center	0.123	0.214	0.329	0.567	1.131	0.743	1.721
CBT	−0.015	0.121	0.015	0.901	0.985	0.778	1.248
Better Access to Patient Care							
Age	−0.011	0.005	6.303	0.012	0.989	0.980	0.997
Cisgender Woman	0.049	0.104	0.224	0.636	1.051	0.856	1.289
White	0.003	0.175	0.000	0.986	1.003	0.712	1.413
Rural	0.144	0.176	0.673	0.412	1.155	0.818	1.632
Medical Center	0.875	0.248	12.456	0.000	2.399	1.476	3.900
CBT	0.461	0.103	20.107	0.000	1.586	1.297	1.941
Patient Demand							
Age	−0.016	0.004	16.194	0.000	0.984	0.977	0.992
Cisgender Woman	0.145	0.088	2.707	0.100	1.156	0.973	1.375
White	0.377	0.142	7.032	0.008	1.458	1.103	1.927
Rural	−0.276	0.140	3.897	0.048	0.759	0.577	0.998
Medical Center	0.170	0.162	1.101	0.294	1.186	0.863	1.629
CBT	0.374	0.090	17.348	0.000	1.454	1.219	1.734
Efficient Use of Time							
Age	−0.003	0.004	0.881	0.348	0.997	0.989	1.004
Cisgender Woman	−0.042	0.086	0.237	0.626	0.959	0.811	1.134
White	−0.441	0.145	9.183	0.002	0.644	0.484	0.856
Rural	0.018	0.138	0.018	0.895	1.018	0.778	1.334
Medical Center	0.174	0.151	1.335	0.248	1.190	0.886	1.600
CBT	0.244	0.088	7.793	0.005	1.277	1.076	1.516
Adequate Technology for Telepsychology Use							
Age	0.006	0.004	2.236	0.135	1.006	0.998	1.013
Cisgender Woman	0.073	0.086	0.732	0.392	1.076	0.910	1.273
White	−0.164	0.139	1.398	0.237	0.849	0.646	1.114
Rural	−0.095	0.139	0.473	0.491	0.909	0.693	1.193
Medical Center	0.216	0.148	2.134	0.144	1.240	0.929	1.656
CBT	0.126	0.089	2.022	0.155	1.134	0.953	1.349
Supportive Laws and HIPAA Regulations							
Age	−0.001	0.004	0.092	0.762	0.999	0.991	1.006
Cisgender Woman	0.253	0.091	7.672	0.006	1.288	1.077	1.541
White	0.046	0.148	0.099	0.753	1.048	0.784	1.399
Rural	0.182	0.142	1.643	0.200	1.200	0.908	1.586
Medical Center	−0.485	0.170	8.140	0.004	0.615	0.441	0.859
CBT	0.130	0.094	1.928	0.165	1.139	0.948	1.369
Lower Cost for Patient or Provider							
Age	0.007	0.004	3.037	0.081	1.007	0.999	1.015
Cisgender Woman	−0.172	0.090	3.617	0.057	0.842	0.705	1.005
White	−0.413	0.143	8.398	0.004	0.662	0.500	0.875
Rural	−0.114	0.148	0.592	0.442	0.892	0.667	1.193
Medical Center	0.457	0.151	9.185	0.002	1.579	1.175	2.122
CBT	0.273	0.096	8.124	0.004	1.314	1.089	1.586
Improved Quality of Delivered Care or Effectiveness							
Age	0.006	0.004	1.990	0.158	1.006	0.998	1.014
Cisgender Woman	0.208	0.100	4.360	0.037	1.231	1.013	1.496
White	−0.212	0.154	1.896	0.169	0.809	0.598	1.094
Rural	0.148	0.154	0.931	0.335	1.160	0.858	1.567
Medical Center	0.020	0.170	0.014	0.906	1.020	0.731	1.425
CBT	0.154	0.103	2.253	0.133	1.167	0.954	1.427

## Data Availability

Data are available upon request made to the corresponding author.
